# Survival analysis of COVID-19 patients in Ethiopia: A hospital-based study

**DOI:** 10.1371/journal.pone.0268280

**Published:** 2022-05-09

**Authors:** Abdene Weya Kaso, Gebi Agero, Zewdu Hurissa, Taha Kaso, Helen Ali Ewune, Habtamu Endashaw Hareru, Alemayehu Hailu

**Affiliations:** 1 School of Public Health, College of Medicine and Health Science, Dilla University, Dilla, Ethiopia; 2 Department of Public Health, College of Health Science, Arsi University, Assela, Ethiopia; 3 Department of Internal Medicine, College of Health Science, Arsi University, Assela, Ethiopia; 4 Department of Surgery, College of Health Science, Arsi University, Assela, Ethiopia; 5 Bergen Centre for Ethics and Priority Setting, Department of Global Public Health and Primary Care, University of Bergen, Bergen, Norway; Children´s Hospital of México Federico Gómez, MEXICO

## Abstract

**Background:**

COVID-19 is a global public health problem causing high mortality worldwide. This study aimed to assess time to death and predictors of mortality among patients hospitalized for COVID-19 in the Arsi zone treatment center.

**Method:**

We performed a retrospective observational cohort study using medical records of laboratory-confirmed COVID-19 cases hospitalized at Bokoji Hospital COVID-19 treatment center from 1^st^ July 2020 to 5^th^ March 2021. We extracted data on the patients’ sociodemographic and clinical characteristics from medical records of hospitalized patients retrospectively. We carried out Kaplan Meier and Cox regression analysis to estimate survival probability and investigate predictors of COVID-19 death 5% level of significance. The Adjusted Hazard Ratio (aHR) with 95% Confidence Interval (CI) was estimated and interpreted for predictors of time to death in the final cox model.

**Result:**

A total of 422 COVID-19 patients treated were analyzed, of these more than one tenth (11.14%) deaths, with a mortality rate of 6.35 cases per 1000 person-days. The majority (87.2%) of deaths occurred within the first 14 days of admission, with a median time-to-death of nine (IQR: 8–12) days. We found patients that age between 31 and 45 years (aHR = 2.55; 95% CI: (1.03, 6.34), older than 46 years (aHR = 2.59 (1.27, 5.30), chronic obstructive pulmonary disease (aHR = 4.60, 95%CI: (2.37, 8.91), Chronic kidney disease (aHR = 5.58, 95%CI: (1.70, 18.37), HIV/AIDS (aHR = 3.66, 95%CI: (1.20, 11.10), admission to the Intensive care unit(aHR = 7.44, 95%CI: (1.82, 30.42), and being on intranasal oxygen care (aHR = 6.27, 95%CI: (2.75, 4.30) were independent risk factors increasing risk of death from COVID-19 disease than their counterparts.

**Conclusion:**

The risk of dying due to COVID-19 disease was higher among patients with HIV/AIDS, chronic obstructive pulmonary disease, and chronic kidney diseases. We also found that older people, those admitted to ICU, and patients who received intranasal oxygen care had a higher risk of dying due to COVID-19 disease. Therefore, close monitoring hospitalized patients that are old aged and those with comorbidities after hospitalization is crucial within the first ten days of admission.

## Introduction

Coronavirus disease -19 (COVID-19), caused by severe acute respiratory syndrome coronavirus 2 (SARS-CoV-2), was identified for the first time in China in December 2019 [1]. On March 11, 2020, the World Health Organization (WHO) declared COVID-19 a pandemic [2]. As of 14^th^ March 2022, based on the WHO daily report, the global burden of COVID-19 has reached 435,985,288 confirmed cases and 6,012,324 deaths [3]. Africa has reported 11,432,000 confirmed cases, of these more than 10.5 million were recovered, and 250,887 died. In Ethiopia, until 14^th^ March 2022, there have been 469,184 confirmed cases of COVID-19 with 7,486 deaths, reported to WHO [4, 5].

COVID-19 affects different people in different ways and around 80% of infected people will develop asymptomatic to mild illness and recovered without hospitalization. In addition, evidence from China and high-income countries showed that about 15% and 5% of COVID-19 infected people had severe and critical illnesses respectively. The overall mortality rate is about 0.25%, with higher death and probability of developing critical illness observed among the older-aged group; people who live in a deprived area, and had comorbidities [6–9].

Therefore, further understanding of the epidemiological and clinical progress of COVID-19 is essential in designing effective and efficient prevention and treatment strategies. These types of evidence were much crucial in resource-limited settings where health care resources are meagre. For instance, every patient who tested positive for SARS-COV-2 was quarantined and observed until recovery during the pandemic’s early phases in Ethiopia. However, as the number of cases increased, the admission and discharge criteria were changed to accommodate the service to those who need it most [10]. Unfortunately, few analyses exist on the epidemiological and clinical progress of COVID-19 examining survival outcomes, patient characteristics, and predictive risk factors for critical illness and death in low-income settings [11, 12]. To the best of our knowledge, no analysis has been yet documented from Ethiopia examining the survival of COVID-19 and associated risk factors. Therefore, this study aimed to assess the time-to-death and risk factors of mortality among COVID-19 patients in the Arsi zone.

## Method and material

### Study design, setting, and population

We performed a retrospective observational cohort study using medical records of 422 laboratory-confirmed COVID-19 cases hospitalized at Bokoji Hospital COVID-19 treatment center from July 1, 2020, to March 5, 2021. All patients were tested positive using Real-time reverse Transcriptase Polymerase Chain Reaction (RT-PCR) SARS-CoV-2 assay of a nasopharyngeal swab specimen. This study was conducted in Bokoji Hospital COVID-19 treatment center located in the Arsi zone, 175 km from Addis Ababa (the capital). Arsi zone comprises about 30 districts (28 *Woreda* and two town administrations). The Zone comprises seven hospitals and 104 health centres, with COVID-19 treatment provided in Bokoji Primary hospital. The hospital is located about 230 km from Addis Ababa and 56 km from the Zonal capital city and has 222 health professionals and 101 supportive health workers [13]. All COVID-19 patients admitted from July 1, 2020, to March 5, 2021, were study population, with those who were referred to the nearby treatment center and had incomplete data were excluded.

### Sample size and sampling techniques

We estimated the optimum sample size using the survival analysis formula [14] with the assumption of a 95% confidence level and a study power of 80%, equal number of cases, and COVID-19 mortality rate and Hazard Rate from the previous study [15]. We calculated the number of events (death) appling the formula E = (Zα/2 + Zβ) ^2^/ (log (HR)) ^2^q0q1, where, z α/2 = 1.96, Z_β_ = 0.84, HR = 3.9, q1 = proportion of subjects that are in exposed group and q0 = proportion of subjects that are in unexposed group. According to the calculation, the number of events (death) obtained was 26. The optimum sample size was calculated using the formula (N) = E/PE, where PE is the cumulative mortality rate (6.3%) from a previous study on COVID-19 [15]. The optimum sample size calculated was 413 COVID-19 patients. However, we included 422 COVID-19 patients’ medical records retrospectively from 1^st^ July 1, 2020, to March 5^th^, 2021, using all consecutive sampling in the study setting.

### Study variables and operational definitions

The outcome of interest for this study was time to death since COVID-19 symptom onset. This outcome was binary with “1” corresponding to when the subject had developed an event (died) and “0” corresponding to when the subject was censored. Respondents were assigned to “0” if they were cured, clinical improved, and discharged with consent or transferred to nearby treatment centers. In general, the verification of an event (death or recovery) was established by the physician’s approval of the final status of patients on their medical records. The sociodemographic factors, health-related factors, comorbidity conditions, clinical manifestation, and treatment-related factors were considered independent variables. We defined patients’ health status at admission in line with updated WHO and the Ethiopian COVID-19 national diagnosis and treatment protocol [16, 17]. The status at admission includes 1) Asymptomatic cases, in which the patient had no clinical signs and symptoms but tested positive for SARS-CoV-2 using RT-PCR test; (2) mild cases, in which the patient had mild clinical symptoms and no imaging findings of pneumonia; (3) moderate cases, in which the patient had a fever, respiratory symptoms, and imaging manifestations of pneumonia and SpO2 of 94% on a room at sea level; (4) severe cases, in which the patient had shortness of breath, respiration rate ≥ 30/min; SpO2 ≤ 94% at rest; arterial partial pressure of oxygen (PaO2)/oxygen concentration (FiO2) ≤ 300 mmHg and pulmonary imaging showing lung infiltrate >50% within 24–48 hours; and (5) critical cases in which the patient develops respiratory failure; shock; and multiple organ dysfunction, requiring monitoring and treatment in the intensive care unit. In addition, comorbidity status of COVID-19 patients was defined as patients with at least one known pre-existing medical illness or reported based on clinical investigation of the physician.

### Data collection and quality management

The medical records of hospitalized patients were retrospectively reviewed and relevant data were extracted and collected using two nurses after one-day training on data abstraction form. We extracted sociodemographic characteristics (i.e. age, sex, and residence), and clinical characteristics such as:- date of onset of illnesses, date of admission, status at admission, presence of comorbidities, types of comorbidities, care provided, treatment outcome, and date of discharge from the records of COVID-19 patients using a structured datasheet. The first author (AK) supervised data collection and entry for completeness in real-time.

### Data analysis

Collected data were entered, coded, and analyzed using Statistical Package for Social Science (SPSS) software Version 25. We summarized the continuous variables using mean with Standard deviations (SD) or median with IQR (interquartile Range) whereas categorical variables were described by frequencies, and percentages. We used the Kaplan–Meier method, the Mann-Whitney test, and the log-rank test to calculate the survival time and compare the survival time among different groups of patients. The Cox regression analysis was used to determine factors associated with death among hospitalized COVID-19 patients. We checked the Cox proportional hazard assumptions on significant covariates by Schoenfeld residuals global test and log-log plot and the overall model adequacy of the proportional hazard model was assessed by using the Cox Snell residual graph. We considered covariates with a p-value of less than 0.25 in the bivariate analysis as a potential candidate for multivariate Cox regression analysis. Variables with a P-value of less than 0.05 were considered statistically significant.

### Ethics approval and consent to participate

Arsi University, College of Health Sciences Research Ethics Review Board approved ethical clearance, and the Institutional Review Board (IRB) waived the informed consent procedure for this study. Since the study was conducted through a review of medical records, consent to participate was waived. Individual patients were not harmed, and the data analyzed was used for this study only.

## Results

### Patient’s sociodemographic characteristics and death rate

A total of 422 COVID-19 patients’ records were included in the study, with the mean age of the participants being 41.06 ± 20.61 years. The majority (61.4%) of the participants were males, and around three-fifth (60.4%) of them were from urban areas. Among 47 cases, 30 deaths were observed among males with a death rate of 6.56 cases per 1000 person-days. Moreover, 30 (63.8%) of the total death were also observed among COVID-19 patients who were residing in urban and approximately 59.6% of the event was seen among patients older than 45 years (Table 1).

**Table 1:** Sociodemographic characteristics and treatment outcome status of hospitalized patients with COVID-19 to Bokoji Hospital treatment centre, 2021.

Variable	Total (n = 422) Frequency (%)	Survived (N = 375) Frequency (%)	Death (N = 47) Frequency (%)	Death rate (95% CI) per 1000 person days	Log-rank test, P-value
Sex					
Female	163 (38.6%)	146 (38.9)	17 (36.2)	6.02(3.74,9.68)	0.759
Male	259 (61.4%)	229 (61.1)	30 (63.8)	6.56(4.59,9.39)	
Age (in years)					
<31	168(39.8)	160 (42.7)	8 (17.0)	2.83(1.42,5.66)	0.002
31–45	91 (21.6)	80 (21.3)	11 (23.4)	6.89(3.82,12.44)	
46 and above	163(38.6)	135(36.0)	28 (59.6)	9.41(6.49,13.62)	
Residence					
Urban	255 (60.4)	225 (60.0)	30 (63.8)	6.70(4.69,9.58)	0.632
Rural	167 (39.6)	150 (40.0)	17 (36.2)	5.82(3.62,9.36)	

### Clinical characteristics of COVID 19 patients under treatment

Out of 422 COVID-19 patients, around half (49.05%) had comorbidities, 193(45.7%) received intranasal oxygen care and 12(2.8%) were treated in the Intensive care unit (ICU). In this study, 47 (11.1%) patients were died during follow-up period and the overall mortality rate was 6.35 (95% CI 4.77–8.46) per 1000 person-days. We identified a mean of 9.81 days from onset of symptoms to death. The time from the onset of symptoms to admission was significantly different between died and survived groups (2.29 ±1.46, p <0.001). Among the patients who died, 39(15%) had comorbidities with a death rate of 6.6 cases per 1000 person-days of observation. In addition, 43(91.5%) of the total deaths were seen in COVID-19 patients who received intranasal oxygen care with a mortality rate of 11.27 cases per 1000 person-days. Furthermore, 11(23.4%) of total deaths were observed among patients who were admitted to ICU, with a death rate of 48.67 cases per 1000 person-days. Besides, among people who died, 37(78.7%) were severe cases at admission with a mortality rate of 11.77 cases per 1000 person-days observation (Table 2).

**Table 2:** Clinical characteristics of patients and treatment outcome status of hospitalized with COVID-19 to Bokoji Hospital treatment centre, 2021.

Variable	Total (n = 422) Frequency (%)	Death Frequency (%)	Survived Frequency (%)	Death rate (95% CI) per person-days	P-value
Mortality rate(95%CI)				6.35(4.77,8.46)	
Comorbidities					
Yes	207(49.1)	39(83.0)	168(44.8)	9.97(7.29,13.65)	0.001*
No	215(50.9)	8(17.0)	207(55.2)	2.29(1.15.4.59)	
Hypertension					
Yes	40 (9.5)	6 (14.6)	34 (9.1)	7.70(3.46,17.14)	0.509*
No	382(90.5)	41(85.4)	341(90.9)	6.19(4.56,8.41)	
Cardiovascular disease					
Yes	29 (6.9)	7 (14.9)	22 (5.9)	12.64(6.02,26.51)	0.029*
No	393(93.1)	40(85.1)	353(94.1)	5.85(4.29,7.97)	
Chronic pulmonary disease					
Yes	48 (11.4)	20 (42.6)	28 (7.5)	25.06(16.17,38.85)	0.001*
No	374(88.6)	27(57.4)	347(92.5)	4.09(2.81,5.97)	
Chronic Kidney disease					
Yes	9 (2.1)	4 (8.5)	5 (1.3)	27.78(10.43,74.01)	0.001*
No	413(97.9)	43(91.5)	370(98.7)	5.93(4.40,7.99)	
Asthma					
Yes	35 (8.3)	5 (10.6)	30 (8.0)	7.40(3.08,17.77)	0.589*
No	387(91.7)	42(89.4)	345(92.0)	6.25(4.62,8.46)	
Diabetic Mellitus					
Yes	72 (17.1)	8 (17.0)	64 (17.1)	6.58(4.81,9.01)	0.894*
No	350(82.9)	39(83.0)	311(82.9)	5.4(2.72,10.86)	
HIV/AIDS					
Yes	12(2.8)	6 (12.8)	6 (1.6)	31.58(14.19,70.29)	0.001*
No	410(97.2)	41(87.2)	369(98.4)	5.69(4.19,7.73)	
Malignance					
Yes	15(3.6)	5 (10.6)	10 (2.7)	21.55(8.97,51.78)	0.003*
No	407(96.4)	42(89.4)	365(97.3)	5.86(4.33,7.93)	
Status at Admission					
Asymptomatic/Mild	186(44.1)	4 (8.5)	182 (48.5)	1.49(0.56,3.98)	0.001*
moderate	82(19.4)	6 (12.8)	76 (20.3)	3.81(1.71,8.48)	
Severe	154(36.5)	37(78.7)	117 (31.2)	11.77(8.53,16.24)	
Onset of symptom to admission	2.29 ±1.46	3.96+1.06	2.08+1.37		0.001^b^
Admission to death	5.85±3.25	5.85±2.25			
Onset of symptoms to death	9.81±3.08	9.81±3.08			
Intranasal oxygen use					
Yes	193(45.7)	43(91.5)	150(40.0)	11.56(8.61,15.54)	0.001*
No	229(54.3)	4(8.5)	225(60.0)	0.84(0.27,2.59)	
ICU admission					
No	406(96.2)	36(76.6)	370(98.7)	5.02(3.62,6.96)	0.001*
Yes	16(3.8)	11(23.4)	5(1.3)	48.67(26.96,87.89)	

Note:

*Log-rank test, *P-value*, and ^a^ Mann-Whitney test

### Time of occurrence of death and survival status of COVID- 19 patients

**Fig 1:**
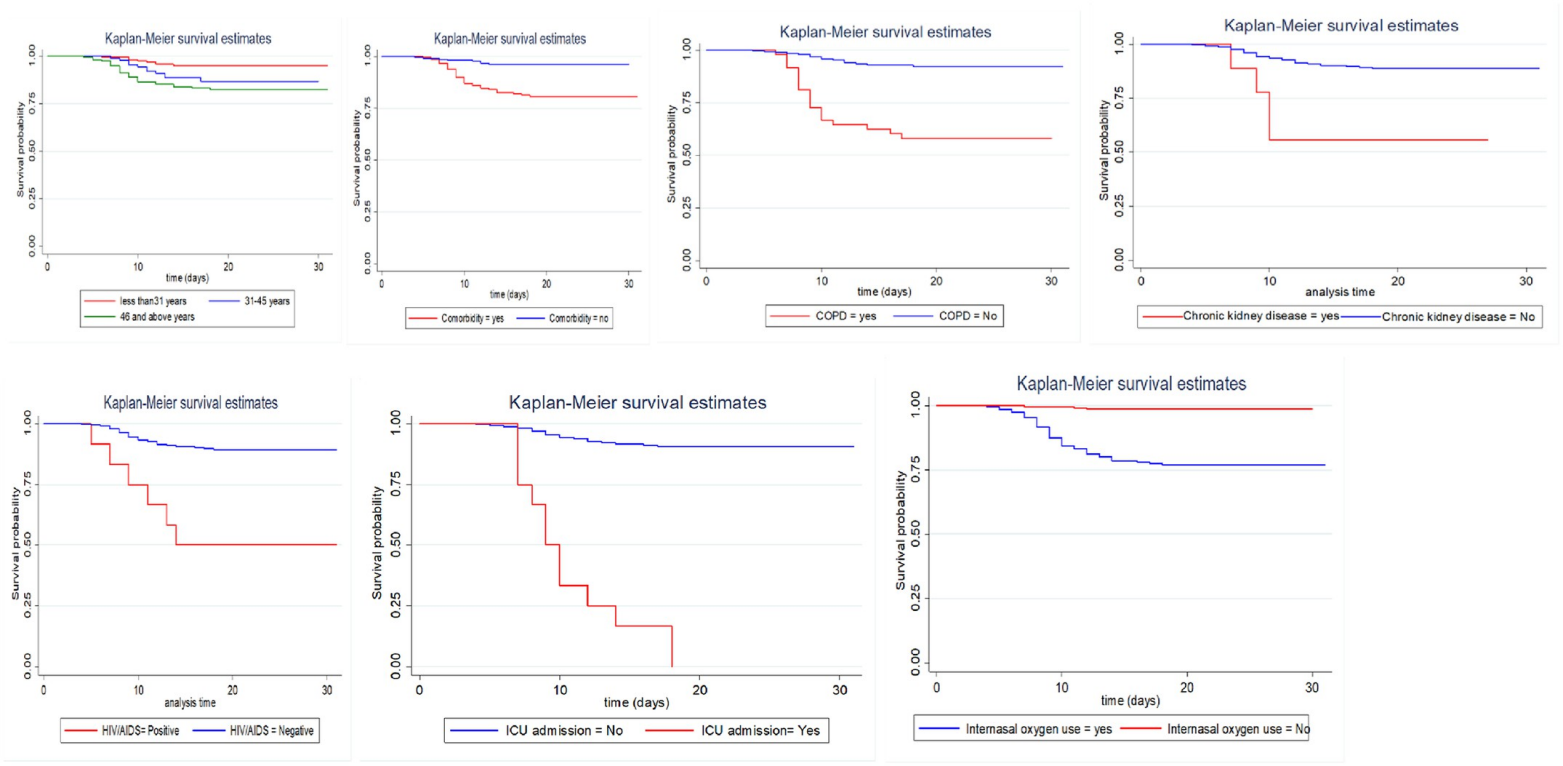
Survival curve of COVID-19 patients hospitalized at Bokoji Hospital treatment centre by sociodemographic and clinical characteristics, 2021.

Of all the COVID-19 deaths, 41 (87.2%) occurred in less than 14 days of admission, and 6 (12.8%) deaths observed on 21 days of admission. Based on the life table estimate, the cumulative survival rate within the first seven days of follow-up was 99% and in the next 7 days was 90%. The median time of death during COVID-19 treatment was 9 days (IQR: 8 days to 12 days) (Table 3). The results of the log-rank test show that the differences in cumulative probability of survival of various factors such as comorbidities, age, chronic obstructive pulmonary disease(COPD), Chronic kidney disease(CKD), Human Immune Virus/Acquired Immune virus Disease Syndrome (HIV/AIDS), ICU admission, and Intranasal Oxygen care were statistically significant (*p <* 0.05), (Fig 1).

**Table 3:** Death rate and survival probability of COVID-19 patients hospitalized at Bokoji Hospital treatment centre, Ethiopia, 2021.

Interval (in days)	# at risk	Death	%	Censored	Survival probability	Cumulative survival probability
1–7 days	422	5	10.6	0	0.99	0.99
8–14 days	389	36	76.6	56	0.91	0.90
15–21 days	224	6	12.8	202	0.97	0.87
22–28 days	69	0	0	96	1	0.87
29–35 days	21	0	0	21	1	0.87

### Predictors of time to death among COVID-19 patients

In the bivariate Cox regression model, we identified potential predictors of time to death at P-value < 0.25. Thus, we considered variables such as age, CKD, COPD, Chronic cardiac diseases, HIV/AIDS, malignancy, intranasal oxygen use, ICU admission, and status at admission for the multivariate Cox regression model. In multivariate Cox regression analysis, survival analysis of hospitalized COVID-19 patients showed that patients with increased age had a higher risk of death in comparison with their counterparts. Patients within the age group of 31–45 and older than 46 years were associated with more than double the risk of mortality (aHR = 2.55; 95% CI: (1.03, 6.34), and 2.59 (1.27, 5.30) respectively. In addition, COPD (aHR = 4.60, 95%CI: (2.37, 8.91); P < 0.001), CKD (aHR = 5.58, 95% CI: (1.70, 18.37); P < 0.005), and HIV/AIDS (aHR = 3.66, 95%CI: (1.20, 11.10); P < 0.022) significantly increased the risk of death in COVID-19 patients. Furthermore, patients admitted to ICU and who received intranasal oxygen had increased risk of death; (aHR = 7.44, 95% CI: (1.82, 30.42); P < 0.005), and (aHR = 6.27, 95% CI: (2.75, 4.30), p<0.001) respectively (Table 4).

**Table 4:** Bivariate and multivariate Cox regression analysis of predictors of death among COVID-19 patients hospitalized at Bokoji Hospital treatment centre, Ethiopia, 2021.

Categories	CHR (95% CI)	aHR (95% CI)	P-value
Age(in years)^a^			
31–45	3.02 (1.34, 6.85)	2.55 (1.03, 6.34)	0.008
46 and above	3.71 (1.69, 8.14)	2.59 (1.27, 5.30)	0.009
Cardiovascular disease	2.37 (1.11, 5.31)	1.69 (0.721,3.98)	0.226
COPD	6.77 (3.79, 12.08)	4.60 (2.37,8.91)	0.001
CKD	5.04 (1.81, 14.08)	5.58 (1.70,18.37)	0.005
Malignance	3.66 (1.45, 9.26)	2.42 (0.858,6.82)	0.095
HIV/AIDS	6.01 (2.55, 14.16)	3.66 (1.20,11.10)	0.022
Intranasal oxygen use	18.02(5.59, 58.09)	7.44(1.82,30.42)	0.005
Status at admission ^b^			
Moderate	3.20 (0.899, 11.34)	1.17 (0.290,4.68)	0.830
Severe	11.30 (4.02, 31.76)	1.24 (0.353,4.36)	0.738
ICU admission	17.62(8.86, 35.04)	6.27(2.75,14.30)	0.001

Note:

^a^ age less than 31 years reference group,

^b^ Asymptomatic/mild reference group,

## Discussion

Our study found that the mortality rate from COVID-19 disease in hospitalized patients was 11.1 (95% CI 8.1–14.2). The overall COVID-19 death rate we found in this study is comparable with findings from India, 8.1% [18], New York, USA, 13.1% [19] and Mexico, 9.37% [20], while it is less than those from Belgium, 29.9% [21], and Italy, 25.2% [22]. This discrepancy between countries might be due to difficulties in conducting diagnosis tests for all suspected individuals, insufficient admission hospital capacity for less severe patients at the early stage of the pandemic and variation in the methodology for cases confirmation and death registery [20, 23, 24].

In our study, the first 14 days of COVID-19 infection were critical, where 87.2% (41) of all deaths occurred. We also found a median time from the onset of symptoms to death of 9 days. This was similar to that reported in China (11days) [24], and different from studies in Mexico(19 days) [15], USA(6 days) [19], and China (18.5days) [25]. This might be explained by differences in method of measuring time to death, study period, strategies for the prevention and control of COVID-19. In Ethiopia, all SARS-CoV2 infected peoples were hospitalized at early stage of the pandemic. However, as the number of cases increased, the admission and discharge criteria were changed to accommodate the service to those who need it most [10]. In multivariate Cox regression, age, being COPD, HIV/AIDS, and CKD patients, admission to ICU, and intranasal oxygen supplementation were statistically significant predictors of time to death. Our study found that the risk of dying from COVID-19 increased with the increasing age of patients. It is in line with previous findings that reported old-aged peoples had an increased risk of death due to COVID-19 disease [25–28]. This is might be due to age-dependent defects in T-cell and B-cell function and an excess production of type 2 cytokines that lead to reduced response in controlling viral replication and prolonged pro-inflammatory responses, potentially leading to death. In addition, a high level of angiotensin-converting enzyme genes in the heart and lungs might also increase the risk of death in this group [15, 25].

We found a high proportion (83%) of people who died from COVID-19 had underlying diseases. The high proportion of comorbidities in those who died from COVID-19 indicates the epidemiological impact of this disease on individuals with chronic illnesses. In our final model, we also found that the risk of dying due to COVID-19 was substantially higher among patients with comorbidities. For instance, patients with chronic obstructive pulmonary disease and chronic kidney disease had an increased risk of death than those without the disease. This finding is in line with a systematic review and meta-analysis of COVID-19 reports and other studies from China, the USA, and Italy indicating that patients with chronic kidney disease and chronic obstructive pulmonary disease were associated with poor prognosis [20, 22, 23, 29, 30]. Likewise, HIV-positive patients with COVID-19 were 3.66 times more likely to die from COVID-19 than HIV-negative individuals in our analysis. The same finding was revealed in a study conducted among patients admitted to Wuhan pulmonary hospital in China [31]. This might be explained by the adverse effects of comorbidities on autoimmune response and metabolic stress that characterizes systemic diseases and decreases the ability to respond against pathogenic agents [25, 32].

In our study, we found a high proportion (78.7%) of individuals who died from COVID-19 disease were in the severe state at admission (P = 738). We also found that patients admitted to ICU and who received intranasal oxygen supplementation had an increased risk of death from COVID-19 infections compared to their counterparts. This finding is consistent with the previous reports [20, 28, 33] that found patients receiving these services had an increased risk of unfavourable treatment outcomes. The observed might be due to patients admitted to ICU and who received intranasal oxygen care being at a high-level of the severity of the disease and deregulated autoimmune response that increased their risk of death. This deregulation of immune response in such patients will be associated with a cytokine storm. Increased amounts of cytokines (i.e. IL-6) are associated with lymphopenia with decreasing CD4+ and CD8+ cell counts, and severe lung injury in patients with COVID-19 that lead to death [34]. This study has some limitations. This study is based on data from a single hospital and may not represent nation pictures. In addition, as this study is based on a retrospective review of patient records, it may not display all factors that may predict variation in survival probabilities.

## Conclusion

In conclusion, the risk of dying due to COVID-19 disease was higher among patients with HIV/AIDS, chronic obstructive pulmonary disease, and chronic kidney diseases. We also found that older people, those admitted to ICU, and patients who received intranasal oxygen care had a higher risk of dying due to COVID-19 disease. Therefore, close monitoring hospitalized patients that are old aged and those with comorbidities after hospitalization is crucial within the first ten days of admission.

## Supporting information

S1 Data (SAV)

## Abbreviations

AHR: Adjusted Hazard Rate
AIDS: Acquired Immune Deficiency Syndrome
ARSD: Acute Respiratory syndrome Distress
CHR: Crude Hazard Rate
CI: Confidence Interval
HIV: Human Immune Virus
HR: Hazard Rate
ICU: Intensive Care Unit
LMIC: Low and Medium-Income Countries
RNA: Ribose Nucleotide Acid
SARS-COV-2: Severe Acute Respiratory Syndrome
SD: Standard Deviation
SPSS: Statistical Package of Social Science Operating
UK: United Kingdom
USA: United States of America
WHO: World Health Organization
